# Captivity Is Associated With Gut Mycobiome Composition in Tibetan Macaques (*Macaca thibetana*)

**DOI:** 10.3389/fmicb.2021.665853

**Published:** 2021-04-16

**Authors:** Binghua Sun, Yingna Xia, Paul A. Garber, Katherine R. Amato, Andres Gomez, Xiaojuan Xu, Wenbo Li, Mingjing Huang, Dongpo Xia, Xi Wang, Jinhua Li

**Affiliations:** ^1^School of Resource and Environmental Engineering, Anhui University, Hefei, China; ^2^International Collaborative Research Center for Huangshan Biodiversity and Tibetan Macaque Behavioral Ecology, Anhui University, Hefei, China; ^3^Department of Anthropology and Program in Ecology, Evolution, and Conservation Biology, University of Illinois, Champaign, IL, United States; ^4^International Centre of Biodiversity and Primate Conservation, Dali University, Dali, China; ^5^Department of Anthropology, Northwestern University, Evanston, IL, United States; ^6^Department of Animal Science, University of Minnesota, St. Paul, MN, United States; ^7^School of Life Science, Hefei Normal University, Hefei, China; ^8^School of Life Science, Anhui University, Hefei, China

**Keywords:** tibetan macaque, captivity, wild, gut mycobiome, diversity

## Abstract

Although recent studies have revealed that gut fungi may play an important functional role in animal biology and health, little is known concerning the effects of anthropogenic pressures on the gut mycobiome. Here, we examined differences of the gut mycobiome in wild and captive populations of Tibetan macaques (*Macaca thibetana*) targeting the fungal internal transcribed spacer (ITS) and using next generation sequencing. Our findings demonstrate that the diversity, composition, and functional guild of the Tibetan macaque gut mycobiome differ across populations living in different habitats. We found that Tibetan macaques translocated from the wild into a captive setting for a period of 1 year, were characterized by a reduction in fungal diversity and an increase in the abundance of potential gut fungal pathogens compared to wild individuals. Furthermore, we found that the relative abundance of two main fungal guilds of plant pathogens and ectomycorrhizal fungi was significantly lower in captive individuals compared to those living in the wild. Our results highlight that, in addition to bacteria, gut fungi vary significantly among individuals living in captive and wild settings. However, given limited data on the functional role that fungi play in the host’s gut, as well as the degree to which a host’s mycobiome is seeded from fungi in the soil or ingested during the consumption of plant and animal foods, controlled studies are needed to better understand the role of the local environment in seeding the mycobiome.

## Introduction

In the face of enormous ecological pressures resulting from human disturbance, nonhuman primates (hereafter NHPs) are experiencing a severe population decline and the threat of extinction on a global scale (Estrada, 2013; Estrada et al., 2017). As an important contributing factor, anthropogenic disturbance often leads to a decrease in the availability and quality of food resources and increased social stress, negatively impacting individuals’ fertility and health (Almeida-Rocha et al., 2017). Recent studies also indicate that anthropogenic disturbance can result in significant changes in host gut microbial community composition including the loss of microbial diversity (Junge et al., 2011; Amato et al., 2013; Barelli et al., 2020). Given that the gut microbiome plays a crucial role in host nutrition, immune systems, and health (Flint et al., 2012; Nicholson et al., 2012; Fung et al., 2017), understanding the effects of anthropogenic disturbance on host gut microbial composition is critical for developing effective conservation strategies for environmental restoration and protecting threatened and declining species (Stumpf et al., 2016; Cavada et al., 2019).

Nonhuman primates that are housed in captive environments (e.g., zoos, rehabilitation and breeding centers) experience changes or restrictions in diet, increased exposure to human-associated microbes, and increased social stress, and are subject to medical interventions including treatments with antibiotics (McKenzie et al., 2017). A number of studies have shown that these changes are associated with the dysbiosis of the host gut microecosystem, including a loss of bacterial diversity (Amato et al., 2013; Clayton et al., 2016; McKenzie et al., 2017; Frankel et al., 2019), a reduction of native gut microbial taxa (Clayton et al., 2016), and an increase in antibiotic resistance genes of the gut microbiome (Tsukayama et al., 2018). Recent studies have shown that providing captive animals with more natural diets or releasing captive animals back into their natural habitat can help restore their native gut bacteria (Schmidt et al., 2019; Liddicoat et al., 2020; Martínez-Mota et al., 2020).

Our understanding of the NHP gut microbiome is based principally on the bacterial component. Less is known about the function and composition of fungal communities in the gut (also known as the mycobiome), especially the effects of environmental change on mycobiome composition. Fungi play critical roles as decomposers, mutualists, and pathogens in a range of ecosystems (Tedersoo et al., 2014). In addition to being a potential pathogen, there is mounting evidence that fungi in the gut serve as an important function in altering gut bacterial composition (Huffnagle and Noverr, 2013; Getzke et al., 2019), modulating host innate and adaptive immune responses (Wüthrich et al., 2012; Rizzetto et al., 2015), and biomass-degrading of host diet (Solomon et al., 2016). In humans, dysbiosis of the gut mycobiome is closely associated with host metabolic and immune disorders, colorectal adenomas, and inflammatory bowel diseases (IBDs; Luan et al., 2015; Mar Rodríguez et al., 2015; Wheeler et al., 2016; Sokol et al., 2017). A recent study found that lab mice released into a natural environment experienced a change in gut mycobiome and an improvement in host immune function (Yeung et al., 2020). Moreover, Barelli et al. (2020) found that the mycobiome community composition differed considerably between primate species (*Procolobus gordonorum* and *Papio cynocephalus*) living in protected and fragmented habitats (Barelli et al., 2020). However, for most primate species, the effects of captivity on host gut mycobiome and its potential impact on host health, remain unknown. In this regard, studies of the gut mycrobiome of individuals living under a range of wild, semi-natural, and captive conditions offer valuable perspectives and tools for investigating and monitoring critical links between diet, environment, and health that can inform captive management, reintroduction programs, and conservation strategies (Stumpf et al., 2016; Trevelline et al., 2019; West et al., 2019).

In the present study, we examined the mycobiome of Tibetan macaques (*Macaca thibetana*), a Near Threatened primate species endemic to China. We compared individuals living in three different environmental settings: captivity (Tong Ling City Zoo), a free-ranging and semi-provisioned group that is visited by tourists (Mt. Huangshan), and a wild group (Mt. Tianhu). In comparing the mycobiome of individuals in each of these three settings, we addressed three main questions: (1) Are there significant differences in gut fungal diversity among captive, semi-provisioned, and wild populations of Tibetan macaques? Secondly (2) What do differences in the composition and ecological function of the gut mycobiome tell us about the role of fungi in the primate digestive tract? and (3) What are the potential factors that shape mycobiome composition in the gut? We present these results in the context of what is known about the mycobiome in other mammals and discuss the potential impacts of captivity on the primate gut mycobiome.

## Materials and Methods

### Study Objects and Samples Collection

This study was carried out at three sites in southern Anhui Province, China, including Mt. Huangshan (HS), Mt. Tianhu (TH), and the Tong Ling City Zoo (TL; Supplementary Table S1). Mt. Huangshan has been a behavioral research and ecotourism center since 1986. Our study group is composed of 60 individuals and represents a free-ranging group that is provisioned three times per day with a total of 6–8 kg of corn. The amount provisioned is approximately 1/3 of the daily food intake of the group. Our second field site, Mt. Tianhu, is located some 10 km from HS. This Tibetan macaque study group was discovered in 2018 and soon thereafter, we began followed and collecting data on this wild group. This group is composed of 91 macaques and obtains all of its diet from the wild. The habitats at both HS and TH are best described as deciduous and evergreen broad-leaf mixed montane forests. Both sites share similar flora and fauna. The main diet of the HS and TH group includes leaves and grass, and to a lesser extent, fruits, flowers, roots, and insects. Corn, which is a major part of the diet of the semi-provisioned group at HS is absent from the diet of the wild group (TH). The group living in captivity (TL) was translocated from HS approximately 1 year prior to our sampling period. The main diet of this group is corn supplemented with sweet potatoes.

Given that seasonal changes in diet can drive variation in the gut mycobiome of Tibetan macaques (Sun et al., 2018); all samples were collected during a 2-week period during the summer, from August 1 to 14, 2019. The monkeys were followed closely by our research team to ensure that each fecal sample collected came from a different individual. In total, we obtained 52 fresh fecal samples of macaques. Twenty-one samples were obtained from the wild, semi-provisioned group (HS), nine from the non-provisioned wild group (TH), and 21 from the captive group (TL). All fecal samples were collected, placed in a sterilized sampling tube with RNAlater (QIA-GEN, Valencia, CA, United States), and transported in ice bags to the laboratory at Anhui University within 12 h of collection. In the lab, samples were stored at −80°C. This research was approved by the Institutional Animal Care and Use Committee of the Anhui Zoological Society (permit number AHZS201711008). We performed all experiments in accordance with their approved guidelines and regulations, and complied with all principles of the China Animal Ethics Committee.

### DNA Extraction and Sequencing

To avoid soil contamination, we extracted the DNA from feces collected from the inner part of each fecal sample using a QIAamp® Fast DNA Stool Mini kit (Qiagen). DNA extraction methods were carried out according to the manufacturer’s instructions. The total DNA extracted from the 52 samples was sent to the Shanghai Majorbio Bio-pharm Technology Co., Ltd. (Shanghai, China) for sequencing. The ITS regions were identified by ITS1F (5'-CTTGGTCATTTAGAGGAAGTAA-3') and ITS2 (2043R; 5'-GCTGCGTTCTTCATCGATGC-3') primers (Bokulich and Mills, 2013). PCR reaction mixtures contained 5–100 ng of DNA template, 1 × GoTaq Green master mix, 1 M MgCl_2_, and 5 pmol of each primer. Reaction conditions consisted of an initial 95°C for 2 min, followed by 35 cycles of 95°C for 30s, 55°C for 30s, and72°C for 60 s, and a final extension of 72°C for 5 min. After the individual quantification step, amplicons were pooled in equal amounts, and pair-end 2 × 300 bp sequencing was performed using the Illlumina Miseq platform (San Diego, CA, United States).

### Bioinformatics and Statistical Analysis

We trimmed raw FASTQ sequencing data for the adaptor sequence and for quality control using the sliding window approach implemented in fastp (v0.19.6; Chen et al., 2018). A window of 50 bp was set to filter the reads with a tail mass value of 20 or less. If the average mass value in the window was lower than 20, the rear bases were removed from the window, and the reads with a tail mass value of 50 bp after quality control were filtered. Those containing *N* bases were removed. We merged overlapping paired-end reads using FLASH (v1.2.7; Magoc and Salzberg, 2011), with the minimum overlap set to 10 bp, the maximum error ratio of overlap area was 0.2, and the number of mismatches barcode allowed was 0. The maximum primer mismatch number was 2. Lastly, we clustered the quality-check of sequences into Amplicon Sequence Variants (ASVs) using DADA2 within Qiime 2 to truncate forward and reverse reads, to denoise the data, and to detect and remove chimeras (Callahan et al., 2016; Bolyen et al., 2019). The Ribosomal Database Project (RDP) classifier was used for taxonomic identification of the ASV sequences (Deshpande et al., 2016), and BLAST searches were conducted using Unite databases.^1^ The sequence data have been deposited in NCBI (project number is PRJNA701719).

The Shannon diversity index (Shannon), ASV richness, and unweighted and weighted UniFrac distance matrices were calculated using Qiime 2 (Bolyen et al., 2019). The FUNGuild v1.0 database,^2^ an open annotation tool for parsing fungal community datasets by ecological guild, was used to assign ecological functions (trophic modes and guilds) to each ASV (Nguyen et al., 2016). For this procedure, we considered all assignments with a confidence score of “probable” or “highly probable.” Genera not represented in the database or with a confidence score of “possible” were classified as undetermined. For more details, see Nguyen et al. (2016).

We tested for normal distributions in alpha diversity indices, relative abundances of dominant phyla, and functional guilds using the Kolmogorov-Smirnov normality test. We used a one-way ANOVA and Tukey’s *post hoc* tests to test for differences across study groups in case of a normal distribution, or a Kruskal-Wallis ANOVA with Dunn’s multiple comparison test in cases of an abnormal distribution. Values of *p* were adjusted using a Bonferroni correction. Principal coordinates analysis (PCoA) was performed with the R packages Made4 and Vegan.^3^ Permutational multivariate ANOVA (PERMANOVA) was used to test for differences in beta diversity (unweighted and weighted UniFrac distance) across our three different macaque groups using the Adonis function in the vegan R package (Chen et al., 2012). Linear discriminant analysis effect size (LEfSe) was used with default options to determine genera enriched in each study group (Segata et al., 2011).^4^ In all analyses, the value of *p* was set at 0.05.

## Results

### General Patterns of the Fungal Profile

**Figure 1:**
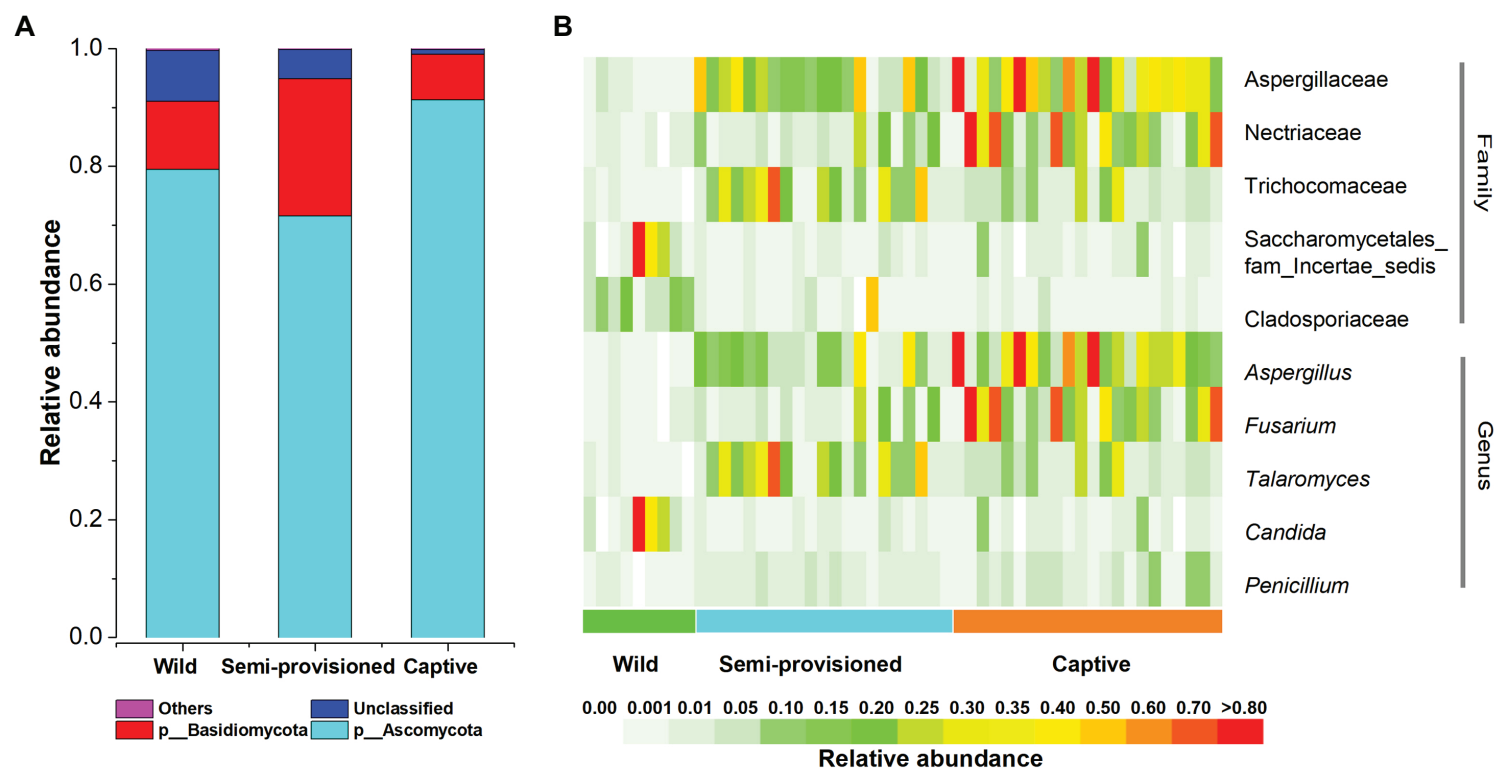
The distributions of phylum, families, and genera. **(A)** Relative abundance of fecal fungal taxa at the phylum level. Stacked bar graphs illustrate the abundances of phyla, and the *x*-axis represents the sample groups. **(B)** The distributions of core families and genera (average relative abundance >0.01, present in more than 90% of all fecal samples).

After bioinformatic processing of 52 Tibetan macaque fecal samples, we obtained 3,067,399 high-quality filtered reads, corresponding to 58,988 ± 3,587 reads per sample. Taxonomic assignment revealed 12 known fungal phyla. The dominant phyla across all samples were Ascomycota (*x* = mean ± *SD*, *x* = 81.24 ± 19.06%) and Basidiomycota (*x* = 14.68 ± 18.34%; Figure 1A). The predominant families were Aspergillaceae (*x* = 25.60 ± 23.56%), Nectriaceae (*x* = 14.57 ± 21.19%), and Trichocomaceae (*x* = 11.35 ± 15.17%). At the genus level, the fecal samples were dominated by *Aspergillus* (*x* = 19.47 ± 22.48%), *Fusarium* (*x* = 14.01 ± 21.46%), and *Talaromyces* (*x* = 11.33 ± 15.18%).

We defined core genera and families as present in more than 90% of fecal samples and at an average relative abundance of >1%. Our results indicated the existence of five core taxa at the family level (Aspergillaceae, Nectriaceae, Trichocomaceae, Cladosporiaceae, and Saccharomycetales_fam_*Incertae_sedis*) and five at the genus level (*Aspergillus*, *Fusarium*, *Talaromyces*, *Candida*, and *Penicillium*; Figure 1B). Summary statistics for these core taxa are provided in Supplementary Table S2.

### Diversity and Composition of the Gut Mycobiome

**Figure 2:**
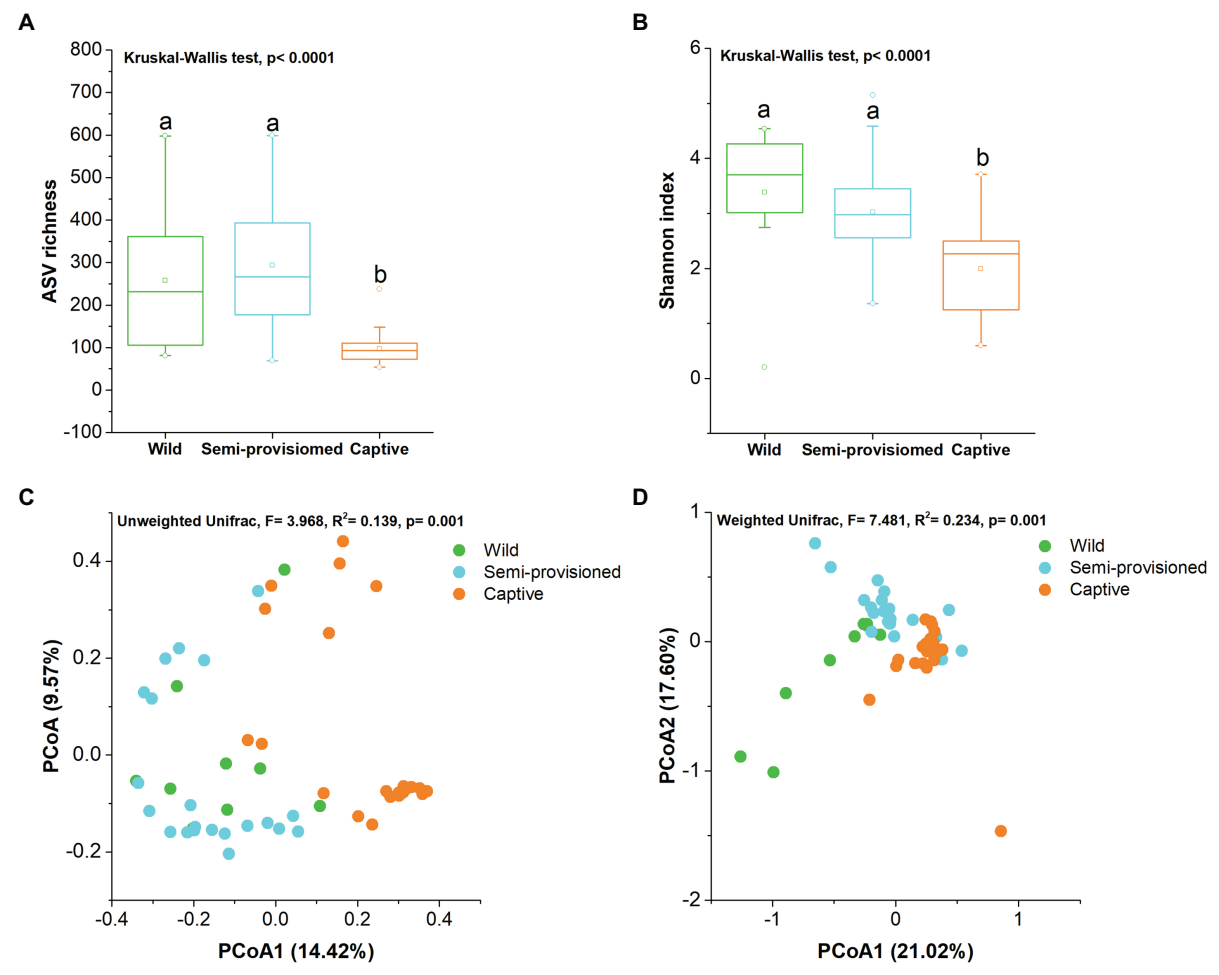
Differences in fecal fungal diversity across three study groups. **(A)** Comparison of amplicon sequence variant (ASV) richness. **(B)** Comparison of Shannon diversity index. A Kruskal-Wallis ANOVA test was used to evaluate the variation across treatment groups. *Post hoc* tests (Dunn’s test) for pairwise comparison tests (values of *p* were adjusted by Bonferroni). **(C,D)** Differentiation of fecal mycobiota structure **(C)** based on unweighted UniFrac distance and **(D)** based on weighted UniFrac distance. Principal coordinates analysis (PCoA) was used to show patterns across three study groups. Adonis tests were performed on unweighted and weighted UniFrac, respectively. Significance was set at the 0.05 level.

We found that the fungal diversity of fecal samples showed significant variation across groups (Kruskal-Wallis, ASV richness: *df* = 2, *F* = 26.178, *p* < 0.0001; Shannon: *df* = 2, *F* = 17.441, *p* < 0.001). Additional pairwise comparison analysis showed that the ASV richness of fecal samples from the captive group was significantly lower than for individuals in the semi-provisioned and wild groups (captive vs. semi-provisioned: *F* = −4.971, adjusted *p* < 0.0001; captive vs. wild: *F* = −2.933, adjusted *p* = 0.008). There were no significant differences between the semi-provisioned and wild groups (adjusted *p* = 1; Figure 2A). We next compared the Shannon index between any two groups. The results indicated that the Shannon index for captive individuals was significantly lower than the indices found for semi-provisioned and wild individuals (captive vs. semi-provisioned: adjusted *F* = 3.343, *p* = 0.002; captive vs. wild: *F* = 3.578, adjusted *p* = 0.001). However, no significant difference was found between individuals in the semi-provisioned and wild groups (adjusted *p* = 0.961; Figure 2B).

**Figure 3:**
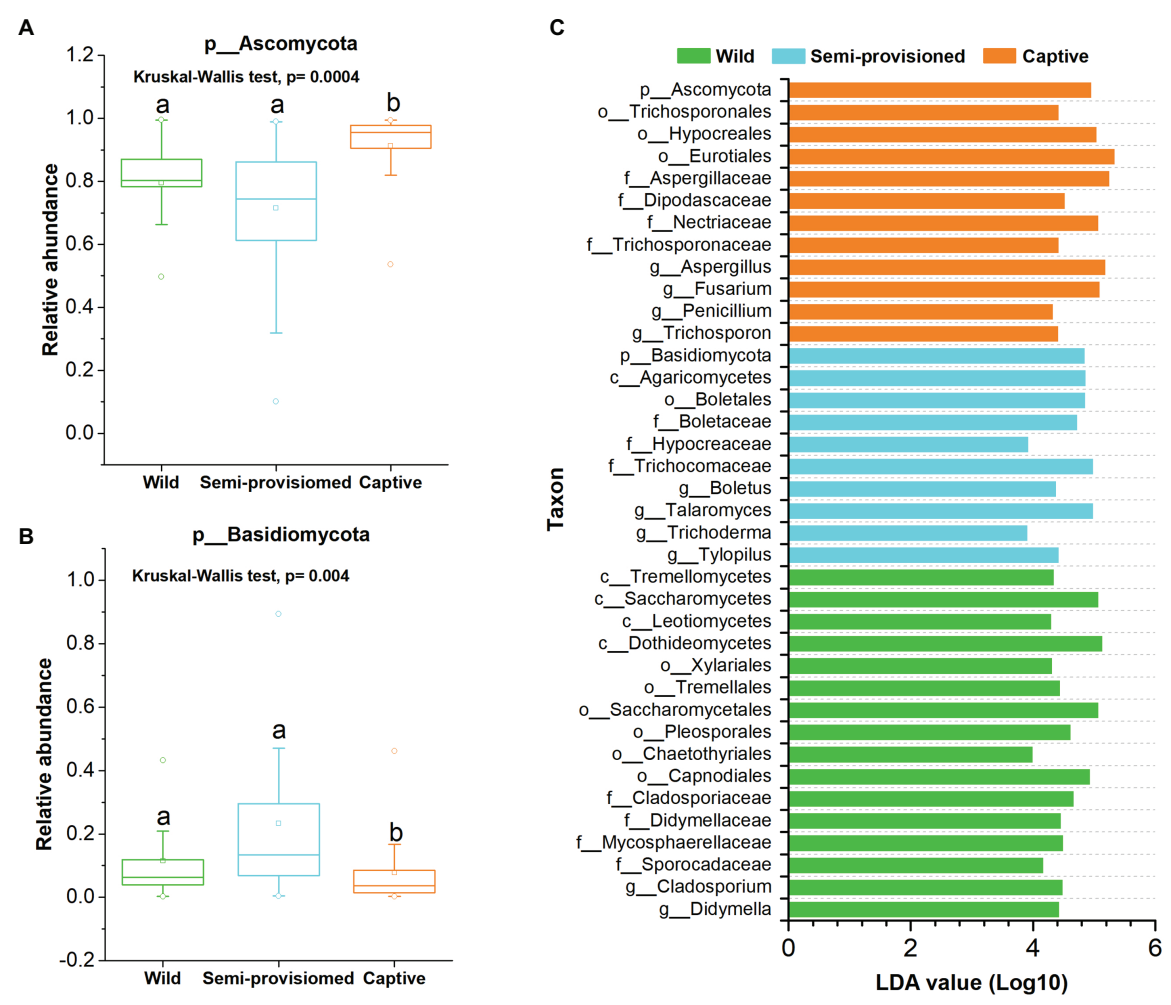
Variation of fecal fungal taxonomy across three study groups. **(A)** Comparison of the dominant phyla Ascomycota. **(B)** Comparison of the dominant phyla Basidiomycota. A Kruskal-Wallis ANOVA test was used to evaluate the variation across treatment groups. *Post hoc* tests (Dunn’s test) for pairwise comparison tests (values of *p* were adjusted by Bonferroni). **(C)** Indicators of known fungal taxa in one of the three groups (at the genus, family, order, class, and phylum levels, the mean relative abundance of known taxa accounting for ≥1% of all the fecal sample), identified by linear discriminant analysis effect size (LEfSe) analysis (LDA > 3, *p* < 0.05).

Principal coordinates analysis and PERMANOVA tests of unweighted and weighted unifrac dissimilarities revealed the variation in the overall taxonomic composition of the fecal mycobiome across samples from the three study groups (PERMANOVA, unweighted, *df* = 2, *F* = 3.968, *R*^2^ = 0.394, *p* = 0.001; weighted, *df* = 2, *F* = 7.481, *R*^2^ = 0.234, *p* = 0.001; Figures 2C,D). Kruskal-Wallis tests revealed that the two dominant fungal phyla showed significant variation across groups (Ascomycota, *df* = 2, *F* = 15.572, *p* = 0.0004; Basidiomycota, *df* = 2, *F* = 11.122, *p* = 0.004; Figures 3A,B). *Post hoc* tests indicated that the relative abundance of Ascomycota was significantly higher in captive individuals than in individuals in the semi-provisioned group (*F* = 3.810, adjusted *p* = 0.0004). There was a trend indicating that the relative abundance of Ascomycota in captive individuals also was higher compared with individuals in the wild group (*F* = 2.387, adjusted *p* = 0.051). The relative abundance of Basidiomycota was significantly higher in semi-provisioned individuals than in captive individuals (*F* = −3.329, adjusted *p* = 0.003). However, the relative abundance of the two phyla does not differ significantly between wild and semi-provisioned Tibetan macaques (Ascomycota, *F* = 1.493, adjusted *p* = 0.406; Basidiomycota, *F* = −0.547, adjusted *p* = 1.00).

Linear discriminant analysis effect size analyses revealed that each study population was characterized by different known fungal taxa. In total, 38 known taxa (at the genus, family, order, class, and phylum levels; the mean relative abundance of known taxa accounting for ≥1% of all the fecal samples) were significantly enriched in one of the three groups (LDA > 3, *p* < 0.05; Figure 3C). Among these taxa, 16, 10, and 12 indicators were identified in wild, semi-provisioned, and captive groups, respectively. We defined the high occurrence indicators (at genus level) as present in more than 90% of fecal samples in its corresponding group. The genus *Cladosporium* was overrepresented in wild samples, and two other known genera, *Trichoderma* and Talaromyces, were overrepresented in the semi-provisioned samples. We also found four known genera that were overrepresented in the captive samples. These were *Trichosporon*, *Penicillium*, *Fusarium*, and *Aspergillus*. Summary statistics for the significantly enriched taxa present in each study group and the functional guilds to which they belong are indicated in Supplementary Table S3.

### Functional Trophic Modes and Guilds of Gut Mycobiome

**Figure 4:**
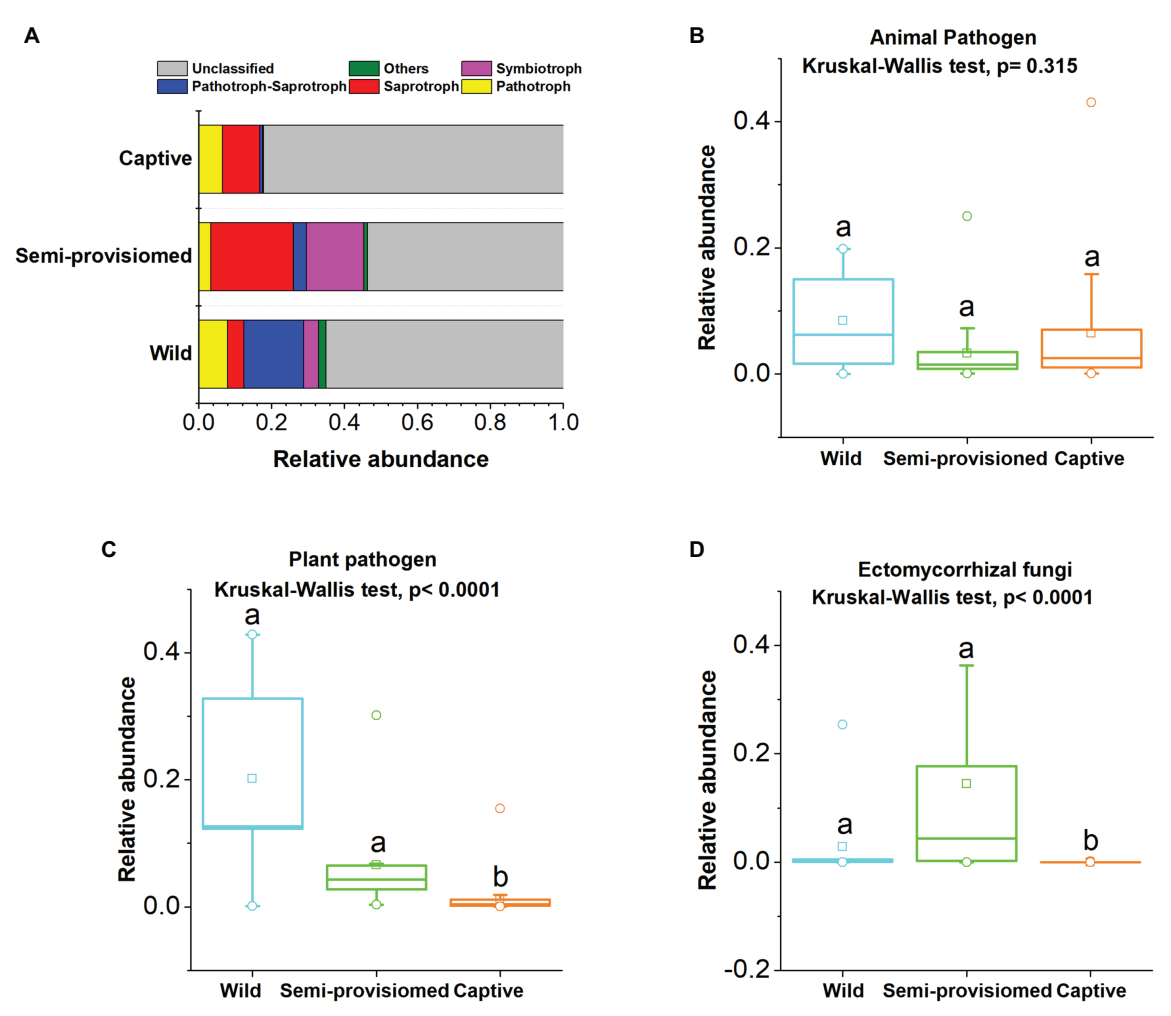
Variation of fecal fungal functional trophic modes across three study groups. **(A)** Relative abundance of functional trophic modes. Stacked bar graphs illustrate the abundances, *x*-axis represents the sample types. **(B**–**D)** Comparison of the functional guilds of animal pathogens, plant pathogens, and ectomycorrhizal fungi, respectively. A Kruskal-Wallis ANOVA test was used to evaluate the variation across treatment groups. *Post hoc* tests (Dunn’s test) for pairwise comparison tests (values of *p* were adjusted by Bonferroni). Significance was set at the 0.05 level.

Based on the FUNGuild v1.0 tool, six functional trophic modes of fecal fungal communities were analyzed. The results indicate that Saprotroph (*x* = mean ± *SD*, *x* = 14.23 ± 15.04%), Pathotroph (*x* = 5.53 ± 7.61%), Pathotroph-Saprotroph (*x* = 4.62 ± 7.99%), and Symbiotroph (*x* = 7.11 ± 17.13%) were the representative and dominant predicted functional trophic modes identified across all samples (Figure 4A). Three main fungal guilds (mean relative abundance was greater than 1%), namely animal pathogens (*x* = 6.69 ± 10.25%), plant pathogens (*x* = 6.64 ± 17.05%), and ectomycorrhizal fungi (*x* = 5.53 ± 8.15%) were detected. The relative abundances of the four functional trophic modes and the three main fungal guilds in each study group (captive, semi-provisioned, and wild) are indicated in Supplementary Table S4.

We found that the two functional guilds of plant pathogens and ectomycorrhizal fungi showed significant variation across the three study groups (Kruskal-Wallis, animal pathogens: *df* = 2, *F* = 2.310, *p* = 0.315; plant pathogens: *df* = 2, *F* = 25.632, *p* < 0.0001; ectomycorrhizal fungi: *df* = 2, *F* = 34.167, *p* < 0.0001; Figures 4B–D). Based on *post hoc* tests, we found that that the relative abundance of the plant pathogen guild was significantly lower in the captive group compared to both the semi-provisioned and wild groups (captive vs. semi-provisioned, *F* = −3.899, adjusted *p* = 0.0003; captive vs. wild, *F* = −4.460, adjusted *p* = 0.0003). Similarly, the guild of ectomycorrhizal fungi was significantly lower among individuals in the captive group compared with individuals in the semi-provisioned and wild groups (captive vs. wild, *F* = −3.171, adjusted *p* = 0.005; captive vs. semi-provisioned, *F* = −5.743, adjusted *p* < 0.0001). However, the relative abundance of two guilds, one for plant pathogens and the other for ectomycorrhizal fungi did not differ significantly between wild and semi-provisioned individuals (plant pathogens, *F* = −1.443, adjusted *p* = 0.447; ectomycorrhizal fungi, *F* = 1.248, adjusted *p* = 0.636).

## Discussion

In the current study, we found that the alpha diversity of the mycobiome of individuals in a captive group of Tibetan macaques translocated from their natural habitat 1 year ago and was reduced compared to individuals in two wild-living groups. One of the wild groups was semi-provisioned. This result is consistent with previous studies on the gut bacterial microbiome of NHPs (Clayton et al., 2016; McKenzie et al., 2017; Frankel et al., 2019). Controlled studies of humans indicate that the loss of gut fungal diversity is associated with susceptibility to diseases, including IBDs (Sokol et al., 2017). In other mammals, a loss of gut fungal diversity has been shown to result in a decrease in the ability to digest cellulose, and a weakend immune response (Akin et al., 1983; Denman and Mcsweeney, 2006; Sokol et al., 2017; Yeung et al., 2020). Therefore, lower alpha diversity among the captive Tibetan macaques has important implications for primate welfare and may be an early indication of negative health consequences resulting from captivity. Future studies are needed to assess the degree to which loss of gut fungal diversity affects the health of captive primates.

Our results also indicated a marked difference in beta diversity between the semi-provisioned group and the completely wild groups, but no difference in alpha diversity, despite the fact that the HS group has several decade of ecotourism and approximately one-third of their diet is provisioned (Li and Kappeler, 2020). This result is consistent with previous finding in two tropical primate species, one living in a protected forest and the other living in a fragmented forest (Barelli et al., 2020). In addition, it has been reported that lab mice exposed to a natural environment to have a more diverse mycobiome and that should provide positive consequences for host’s immune function (Yeung et al., 2020). Our results together with recent finding revealed that living in the wild and consuming a diverse diet are beneficial to maintaining gut fungal diversity of NHPs, while living in captivity, consuming a less varied diet, and limited exposure to soils and wild plants are expected result in a significant reduction in gut fungal diversity.

Previous studies have implied that fiber-rich diets may stimulate gut fungal diversity. For example, in ruminants, individuals consuming a diet high in plant fiber were found to exhibit higher gut fungal diversity compared with individuals fed a diet lower in plant fiber, such grains (Denman and Mcsweeney, 2006; Denman et al., 2010; Boots et al., 2013). Although, we do not have quantitative data on the fiber content of the wild plant foods consumed by the wild and semi-provisioned macaques, they naturally consume a broad-based diet that included grasses, leaves, fruits, and insects (Huffman et al., 2020). However, the main diet of captive group was corn supplemented with sweet potatoes. Corn fed to the captive macaques contains approximately 9 g of dietary fiber per 100 g consumed, and fiber in sweet potatoes contains approximately 10 g of dietary fiber per 100 g consumed (Sun et al., 2014). A previous study revealed that the crude fiber content of the major components of the plant diet of wild living Tibetan macaques ranged from 17.91 to 47.92% (You et al., 2013). Lower dietary fiber in the foods consumed by the captive macaques offers a possible explanation for their decreased gut mycobiome diversity compared to the wild and semi-provisioned free-ranging macaque population.

In addition to diet, other environmental sources of gut fungi include the soil (Fiers et al., 2019; Richard and Sokol, 2019; Mann et al., 2020). Given the conditions of their captive environment, which included frequent cleaning of concrete surfaces and metal bars, access to soil fungi was extremely limited. In this regard, we found that the relative abundance of the two main fungal guilds of plant pathogens and ectomycorrhizal fungi was decreased significantly in captive individuals compared to individuals living in both wild groups. Plant pathogenic and ectomycorrhizal fungi are two dominant guilds naturally found in plant and soil (Averill et al., 2016; Yang et al., 2019). Although our data showed a marked difference in mycobiome composition between the semi-provisioned free-ranging group and the un-provisioned wild group, mycobiome diversity between these groups did not differ. This pattern suggests that the free-ranging group was not exposed to fewer fungi but rather than differences in the environment and diet resulted in exposure to distinct fungi and/or altered selective pressure on the existing fungal community in the macaque gut.

We also found that the gut mycobiome of macaques in all three study groups was dominated by two phyla Ascomycota and Basidiomycota. This is consistent with previous studies in humans and NHPs (Hallen-Adams et al., 2015; Strati et al., 2016; Suhr et al., 2016; Wheeler et al., 2016; Sokol et al., 2017; Sun et al., 2018). Our results demonstrated that several fungal taxa differed in relative abundance, at the levels of genus, family, order, class, and phylum, among our three study groups. As described for fungal diversity, this finding may be a result of diet since recent studies of the human gut mycobiota have found the abundance of gut fungal taxa fluctuates with host diet. For example, plant-based diets (rich in grains, legumes, fruits, and vegetables) were associated with enrichment in *Candida*, whereas individuals consuming animal-based diets (rich in meats, eggs, and cheeses) were enriched in *Debaryomyces* and *Penicillium* (David et al., 2014; Hallen-Adams and Suhr, 2017). In particular, the zgenus *Trichosporon* (relative abundance, 5.82%; occurrence rate 100%) was assigned to the guild of potential animal pathogens based on FUNGuild analyses. Several species in this genus are responsible for the majority of cases of trichosporonosis, which are important opportunistic pathogens of humans (Marty et al., 2003; Colombo et al., 2011). However, although captivity may increase disease risk in primates, it is unclear if captive Tibetan macaques in this study showed a compromised immune function.

Interestingly, the genera *Penicillium* and *Aspergillus*, which were overrepresented in the captive individuals, also were enriched in the semi-provisioned free-ranging individuals compared to the wild individuals. These two genera, commonly reported as core genera in human gut mycobiome studies, also are found in foods consumed by humans and prone to spoilage (Suhr and Hallen-Adams, 2015; Hallen-Adams and Suhr, 2017). Although varying in degree, corn was a major component of the diet of both the captive and semi-provisioned free-ranging populations. Thus, we speculate either that a corn-derived fungi or possible transmission of gut fungi between humans handling the corn and the monkeys, may have promoted the increase of these two fungal genera.

Tibetan macaques in the wild and semi-provisioned groups had access to fungi naturally occurring in the soil and in animals and plants consumed. We found that the two main fungal guilds of plant pathogen and ectomycorrhizal fungi, which are commonly reported in plant or soil mycobiome, were decreased significantly in captive individuals (Averill et al., 2016; Yang et al., 2019). In addition, *Cladosporium* which was overrepresented in the wild group, and *Trichoderma* and *Talaromyces*, which characterized the semi-provisioned free-ranging group, have been isolated from soil and organic matter such as decaying wood and fruit (Potin et al., 2004; Chen and Zhuang, 2017; Salomão, 2018). Therefore, the presence and availability of fungi associated with foods and soil in the natural environment offer a likely explanation for certain specific differences in the gut fungal composition of wild and free-ranging macaques compared to captive individuals (Fiers et al., 2019; Richard and Sokol, 2019; Mann et al., 2020). However, a lack of information about metabolic pathways characterizing gut fungi and the influence of diet and environment in mycobiome composition and function, impact our ability to fully interpret these results.

## Conclusion

Our findings demonstrate that individuals translocated from their natural habitat and fed a highly monotonous diet in captivity for 1 year, were characterized by reduced diversity and increased potential fungal pathogens in their gut. Future studies should focus on investigating whether the predominant gut fungal taxa detected here are food-borne travelers or symbiotic residents under modulation by the host and diet. To that end, culture-dependent and metagenomic approaches, along with mapping interactions between gut fungi and gut function are warranted. Additionally, it is essential to fully characterize the dietary behaviors and environmental exposures of primate populations to infer seeding mycobiome sources and the potential impact of gut fungi on primate health. Such data can be used to devise management strategies that can modulate the mycobiome of wild NHPs living in degraded habitats, and the health and longevity of captive primates that may be reintroduced into the wild.

## Data Availability Statement

The datasets presented in this study can be found in online repositories. The names of the repository/repositories and accession number(s) can be found at: NCBI, PRJNA701719.

## Author Contributions

BS and JL conceived and designed the experiments. BS, YX, XX, WL, MH, XW, and DX performed the experiments. BS, YX, XX, PG, KA, and AG contributed to reagents, materials, and analysis tools. BS, YX, XX, PG, KA, AG, and JL wrote the paper. All authors reviewed the manuscript and agreed to the published version of the manuscript.

### Conflict of Interest

The authors declare that the research was conducted in the absence of any commercial or financial relationships that could be construed as a potential conflict of interest.

## Footnotes

^1^https://unite.ut.ee/repository.php

^2^https://github.com/UMNFuN/FUNGuild

^3^http://www.cran.r-project.org/package=vegan

^4^http://huttenhower.sph.harvard.edu/lefse/
