# Supplementary material for: Captivity Is Associated With Gut Mycobiome Composition in Tibetan Macaques (Macaca thibetana)
Source: Front Microbiol. 2021 Apr 16;12:665853. doi: 10.3389/fmicb.2021.665853 (PMC8085381; doi:10.3389/fmicb.2021.665853)
Supplement: Supplementary file 1 [file Table_1.doc]

**Differences in the gut mycobiome of wild and captive populations of Tibetan macaques (*Macaca thibetana*) - *Supplementary material***

Binghua Sun^1,2^*, Yingna Xia^1,2^, Paul A. Garber^3,4^, Katherine R. Amato^5^, Andres Gomez^6^, Xiaojuan Xu^1,7^, Wenbo Li^1,2^, Mingjing Huang^1,2^, Dongpo Xia^2,8^, Xi Wang^1,2^ and Jinhua Li^1,2,7^*

**Affiliation:**

1 School of Resource and Environmental Engineering, Anhui University, Hefei, China.

2 International Collaborative Research Center for Huangshan Biodiversity and Tibetan Macaque Behavioral Ecology, Anhui University, Hefei, China.

3 Department of nthropology and Program in Ecology, Evolution, and Conservation Biology, University of Illinois, Champaign, IL, United States.

4 International Centre of Biodiversity and Primate Conservation, Dali University, Dali, China.

5 Department of Anthropology, Northwestern University, Evanston, IL, United States. 6 Department of Animal Science, University of Minnesota, St. Paul, MN, United States.

7 School of Life Science, Hefei Normal University, Hefei, China.

8 School of Life Science, Anhui University, Hefei, China.

***Table S1. Information on study sites and samples. TH is approximately 10 kilometers from HS. The captive group is TL. Individuals were translocated from HS about 1 year prior to our sampling period.***

| **Study site** | | **Group type** | **Main Food type** | **Living state** | **Sample type** | **Sample number** |
| --- | --- | --- | --- | --- | --- | --- |
| **Full name** | **Abbreviation** |
| Mt. Huangshan | HS | Semi-provisioned | wild diet + corn | Free-ranging | Fecal | 21 |
| Mt. Tianhu | TH | Wild | wild diet | wild | Fecal | 9 |
| Tong ling City Zoo | TL | Captive | corn+ sweet potatoes | Captivity | Fecal | 22 |

***Table S2.*** The core genera and families of the gut mycobiome across all fecal samples. We defined core genera and families as present in more than 90% of fecal samples and at an average relative abundance of >1%.

| **Taxon** | **Mean relative abundance (all samples)** | **Occurrence rate (all samples)** | **Mean relative abundance (each group)** | | |
| --- | --- | --- | --- | --- | --- |
|  |  |  | **Wild** | **Semi-provisioned** | **Captive** |
| f_Aspergillaceae | 0.256 | 100.0% | 0.549 | 0.223 | 0.387 |
| f_Nectriaceae | 0.146 | 98.1% | 0.439 | 0.069 | 0.270 |
| f_Trichocomaceae | 0.113 | 98.1% | 0.503 | 0.196 | 0.079 |
| f_Cladosporiaceae | 0.036 | 98.1% | 0.521 | 0.038 | 0.007 |
| f_Saccharomycetales_fam_  Incertae_sedis | 0.050 | 94.2% | 0.394 | 0.013 | 0.020 |
| g_*Aspergillus* | 0.195 | 98.1% | 0.003 | 0.137 | 0.328 |
| g_*Fusarium* | 0.140 | 98.1% | 0.006 | 0.063 | 0.268 |
| g_*Talaromyces* | 0.113 | 98.1% | 0.004 | 0.196 | 0.079 |
| g_*Candida* | 0.050 | 96.2% | 0.208 | 0.013 | 0.020 |
| g_*Penicillium* | 0.032 | 100.0% | 0.008 | 0.024 | 0.050 |

***Table S3.*** The known taxa enriched in Tibetan macaque fecal samples from three groups (wild, semi-provisioned and captive). LEfSe analysis was used with the default options in this study, the genera with an LDA score >3 and p<0.05 are indicated in this table. Taxonomic abbreviations: p, phylum; c, class; o, order; f, family; g, genus.

| **Taxon** | **Group** | **LDA value** | **P value** | **Occurrence rate** | **Mean relative abundance** |
| --- | --- | --- | --- | --- | --- |
| g__*Didymella* | Wild | 4.440 | < 0.0001 | 88.9% | 0.050 |
| g__*Cladosporium* | Wild | 4.501 | 0.0017 | 100.0% | 0.065 |
| f__Sporocadaceae | Wild | 4.182 | < 0.0001 | 88.9% | 0.028 |
| f__Mycosphaerellaceae | Wild | 4.509 | < 0.0001 | 100.0% | 0.066 |
| f__Didymellaceae | Wild | 4.469 | < 0.0001 | 88.9% | 0.055 |
| f__Cladosporiaceae | Wild | 4.680 | 0.0008 | 100.0% | 0.101 |
| o__Capnodiales | Wild | 4.950 | < 0.0001 | 100.0% | 0.187 |
| o__Chaetothyriales | Wild | 4.014 | < 0.0001 | 100.0% | 0.021 |
| o__Pleosporales | Wild | 4.628 | < 0.0001 | 100.0% | 0.090 |
| o__Saccharomycetales | Wild | 5.085 | < 0.0001 | 100.0% | 0.272 |
| o__Tremellales | Wild | 4.461 | < 0.0001 | 100.0% | 0.062 |
| o__Xylariales | Wild | 4.327 | < 0.0001 | 88.9% | 0.043 |
| c__Dothideomycetes | Wild | 5.147 | < 0.0001 | 100.0% | 0.293 |
| c__Leotiomycetes | Wild | 4.313 | < 0.0001 | 100.0% | 0.041 |
| c__Saccharomycetes | Wild | 5.085 | < 0.0001 | 100.0% | 0.272 |
| c__Tremellomycetes | Wild | 4.354 | 0.0306 | 100.0% | 0.065 |
| g__*Tylopilus* | Semi-provisioned | 4.437 | 0.0185 | 23.8% | 0.059 |
| g__*Trichoderma* | Semi-provisioned | 3.922 | < 0.0001 | 100.0% | 0.017 |
| g__*Talaromyces* | Semi-provisioned | 4.997 | < 0.0001 | 100.0% | 0.196 |
| g__*Boletus* | Semi-provisioned | 4.396 | 0.0004 | 47.6% | 0.048 |
| f__Trichocomaceae | Semi-provisioned | 4.998 | < 0.0001 | 100.0% | 0.196 |
| f__Hypocreaceae | Semi-provisioned | 3.940 | < 0.0001 | 100.0% | 0.018 |
| f__Boletaceae | Semi-provisioned | 4.737 | < 0.0001 | 81.0% | 0.114 |
| o__Boletales | Semi-provisioned | 4.866 | < 0.0001 | 81.0% | 0.155 |
| c__Agaricomycetes | Semi-provisioned | 4.878 | < 0.0001 | 100.0% | 0.160 |
| p__Basidiomycota | Semi-provisioned | 4.864 | 0.0038 | 100.0% | 0.233 |
| g__*Trichosporon* | Captive | 4.426 | < 0.00001 | 100.0% | 0.058 |
| g__*Penicillium* | Captive | 4.342 | 0.0038 | 100.0% | 0.050 |
| g__*Fusarium* | Captive | 5.108 | < 0.0001 | 100.0% | 0.268 |
| g__*Aspergillus* | Captive | 5.202 | < 0.0001 | 100.0% | 0.328 |
| f__Trichosporonaceae | Captive | 4.436 | < 0.0001 | 100.0% | 0.060 |
| f__Nectriaceae | Captive | 5.086 | 0.0006 | 100.0% | 0.270 |
| f__Dipodascaceae | Captive | 4.537 | < 0.0001 | 95.5% | 0.067 |
| f__Aspergillaceae | Captive | 5.267 | < 0.0001 | 100.0% | 0.387 |
| o__Eurotiales | Captive | 5.355 | < 0.0001 | 100.0% | 0.467 |
| o__Hypocreales | Captive | 5.054 | 0.0047 | 100.0% | 0.292 |
| o__Trichosporonales | Captive | 4.436 | < 0.0001 | 100.0% | 0.060 |
| p__Ascomycota | Captive | 4.967 | 0.0004 | 100.0% | 0.912 |

***Table S4.*** Relative abundance of fungal functional guilds in each study group. Only the confidence values considered high probability and probable were counted as known trophic modes or guilds. Cases in which confidence was possible and undefined were defined as unknown trophic modes. Guilds were filtered by the criteria of mean relative abundance greater than 1% in at least one of the three study groups.

| **Functional Guild** | **Wild** | | | **Semi-provisioned** | | | **Captive** | | |
| --- | --- | --- | --- | --- | --- | --- | --- | --- | --- |
|  | **N** | **Mean** | **SD** | **N** | **Mean** | **SD** | **N** | **Mean** | **SD** |
| **Trophic mode** |  |  |  |  |  |  |  |  |  |
| Pathotroph | 9 | 0.0802 | 0.0724 | 21 | 0.0338 | 0.0247 | 22 | 0.0657 | 0.1032 |
| Saprotroph | 9 | 0.0447 | 0.0272 | 21 | 0.2263 | 0.1871 | 22 | 0.1021 | 0.0921 |
| Pathotroph-Saprotroph | 9 | 0.1640 | 0.1079 | 21 | 0.0356 | 0.0571 | 22 | 0.0082 | 0.0198 |
| Symbiotroph | 9 | 0.0400 | 0.0845 | 21 | 0.1580 | 0.2412 | 22 | 0.0008 | 0.0030 |
| Others | 9 | 0.0205 | 0.0283 | 21 | 0.0101 | 0.0113 | 22 | 0.0018 | 0.0030 |
| Unclassified | 9 | 0.7308 | 0.1343 | 21 | 0.5700 | 0.2358 | 22 | 0.8871 | 0.0935 |
| Pathotroph | 9 | 0.0802 | 0.0724 | 21 | 0.0338 | 0.0247 | 22 | 0.0657 | 0.1032 |
| **Guild** |  |  |  |  |  |  |  |  |  |
| Plant pathogen | 9 | 0.2014 | 0.1542 | 21 | 0.0657 | 0.0698 | 22 | 0.0131 | 0.0325 |
| Ectomycorrhizal | 9 | 0.0308 | 0.0839 | 21 | 0.1513 | 0.2415 | 22 | 0.0001 | 0.0003 |
| Animal Pathogen | 9 | 0.0845 | 0.0759 | 21 | 0.0329 | 0.0541 | 22 | 0.0647 | 0.1010 |
